# The Relevance of Preventive Implantable Cardioverter-Defibrillators (ICDs) in Non-Ischemic Cardiomyopathy in the Presence of Effective Quadruple Therapy for Heart Failure: A Review of Contemporary Medical Literature

**DOI:** 10.7759/cureus.38268

**Published:** 2023-04-28

**Authors:** Mukosolu F Obi, Vikhyath Namireddy, Maria A Reinberg Palmar, Manjari Sharma, Jennifer A Gulas

**Affiliations:** 1 Internal Medicine, Wyckoff Heights Medical Center, New York City, USA; 2 Medicine, St. George's University School of Medicine, True Blue, GRD

**Keywords:** cardiac sudden death, ischemic dilated cardiomyopathy, guideline directed medical therapy, heart failure, implantable cardioverter-defibrillator (icd)

## Abstract

Cardiomyopathy is a disease of the cardiomyocytes that affects their structural function, leading to heart failure (HF). Non-ischemic cardiomyopathy (NICM) includes a subtype of dilated cardiomyopathy (DCM), restrictive cardiomyopathy (RCM), and hypertrophic cardiomyopathy (HCM). These types of cardiomyopathies have no coronary artery vessel involvement. The most common cause of NICM is DCM. In the ischemic cardiomyopathy (ICM) subtype, the utilization of implantable cardioverter-defibrillators (ICDs) has been effective in the prevention of sudden cardiac death (SCD). However, the relevance of ICDs in patients with NICM having an ejection fraction (EF) ≤35%, who are also receiving effective quadruple therapy (i.e., angiotensin-converting enzyme (ACE) inhibitors or angiotensin receptor blockers (ARB), beta-blockers, mineralocorticoid receptor antagonists, and sodium-glucose cotransporter-2 (SGLT2) inhibitors) for HF has been a topic of debate. The purpose of this review is to analyze the benefits of preventive ICDs in NICM patients on adequate quadruple therapy for HF. The current guidelines recommend ICD implantation in patients with NICM who have a left ventricular ejection fraction of ≤35%, come under the New York Heart Association (NYHA) class II or III, and are in sinus rhythm with optimal medical therapy. The evidence supporting this recommendation is limited. Numerous clinical studies and meta-analyses have been conducted to look into this issue. While some have discovered a substantial decrease in mortality with the implantation of an ICD in patients with NICM, others have not found significant changes. Thereby, further investigations are required to define the function of ICDs in this population.

## Introduction and background

Heart failure (HF) is a chronic and progressive disease characterized by the inability of the heart to pump blood effectively [1]. The primary goal of HF management is to improve the quality of life and reduce mortality. Morbidity and mortality have been reduced significantly in HF patients with the induction of quadruple therapy, consisting of an angiotensin-converting enzyme (ACE) inhibitor or an angiotensin receptor blocker (ARB), a beta-blocker, a mineralocorticoid receptor antagonist, and a sodium-glucose cotransporter-2 (SGLT2) inhibitor [2,3].

Implantable cardioverter-defibrillators (ICDs) are implanted in patients at risk of sudden cardiac death (SCD), including those with non-ischemic cardiomyopathy (NICM). Among the subtypes of NCIM, dilated cardiomyopathy (DCM) is the most common type. DCM occurs due to eccentric enlargement of the ventricles, thereby causing an impaired pumping function. This is referred to as heart failure with reduced ejection fraction (HFrEF). Genetic, viral, alcoholic, tachycardia, and postpartum etiologies attribute to this type of HF. Hypertrophic cardiomyopathy (HCM) is a type of NICM caused by concentric thickening of heart muscles in the ventricles, leading to impaired blood flow and arrhythmias as a consequence. HCM initially presents as heart failure with preserved ejection fraction (HFpEF) and is caused by genetic mutations. The ejection fraction (EF) is generally >40% in the early stages. Restrictive cardiomyopathy (RCM) is a rare type of NICM caused due to infiltration of the myocardium leading to impaired ventricle relaxation during the filling of the heart chambers. Like HCM, RCM also initially presents as HFpEF, with an EF >40%. It is caused by conditions like amyloidosis, hemochromatosis, sarcoidosis, and Fabry disease in adults. SCD is a major cause of death in patients with NICM [1,3]. However, the use of ICDs in NICM remains controversial. Several randomized controlled trials have evaluated the use of ICDs in patients with NICM with varying results.

## Review

This review aims to explore the contemporary medical literature on the relevance of preventive implantable cardioverter-defibrillators (ICDs) in the presence of effective quadruple therapy for heart failure in patients with non-ischemic cardiomyopathy (NICM). By examining the latest evidence, this review will provide a comprehensive assessment of the benefits and limitations of preventive ICDs in this patient population and will offer insights into the appropriate use of ICDs in patients with NICM.

The inclusion criteria for this review included literature that evaluated the use of ICD in patients with heart failure (HF), particularly those with the NICM subtype. A search of PubMed and Google Scholar yielded several articles related to this topic. A literature review of these cases, evaluating the New York Heart Association (NYHA) classification of HF, use of quadruple therapy, ICD placement, and cardiac resynchronization therapy with a defibrillator (CRT-D), was conducted (Table 1).

**Table 1:** Results of the literature review conducted. HF: heart failure, ICD: implantable cardioverter-defibrillator, NICM: non-ischemic cardiomyopathy, CRT-D: cardiac resynchronization therapy with a defibrillator, ACE: angiotensin-converting enzyme, NYHA: New York Heart Association, LVEF: left ventricle ejection fraction, SCD: sudden cardiac death, EF: ejection fraction, MI: myocardial infarction, CHF: congestive heart failure.

Authorship and Year of Publication	Objective	Subtype of HF	Therapy	Conclusion
Desai et al. (2004) [4]	To determine whether ICD therapy reduces all-cause mortality in patients with HF	NICM	ICD or CRT-D vs quadruple therapy alone	ICD therapy appears to significantly reduce mortality in selected patients with NICM
Kadish et al. (2004) [5]	To determine whether ICD therapy reduces all-cause mortality in patients with HF	NICM	Standard medical therapy vs standard medical therapy + single-chamber ICD	ICD + ACE inhibitors and beta-blockers significantly reduce the risk of SCD from arrhythmia
Bardy et al. (2005) [6]	To determine whether conventional therapy + ICD vs conventional therapy + amiodarone improves prognosis in patients with HF	NYHA class II or III CHF and an LVEF of 35% or less	Conventional therapy + ICD vs conventional therapy + amiodarone	Amiodarone has no favorable effect on survival, whereas single-lead, shock-only ICD therapy reduces overall mortality by 23%
Kober et al. (2016) [7]	To evaluate the benefit of prophylactic ICDs in patients with systolic HF due to NICM	Symptomatic systolic heart failure (LVEF ≤35%) not caused by coronary artery disease	Standard conventional therapy + CRT vs CRT + ICD	Prophylactic ICD implantation was not associated with a significantly lower long-term rate of death from any cause compared to the clinical care
Shen et al. (2017) [8]	Rates of SCD in patients with HF and reduced EF	NICM	ICD + standard medical therapy vs standard medical therapy alone	ICDs may not significantly reduce overall mortality when added to appropriate medical therapy
Steinberg et al. (2014) [9]	To determine overall mortality for patients receiving an ICD versus no ICD by the extent of medical comorbidity	NICM	ICD vs no ICD placement	Patients with extensive comorbid medical illnesses may experience less benefit from primary prevention ICDs than those with less comorbidity
Goldenberg et al. (2014) [10]	To evaluate the effects of CRT-D in the long-term survival of heart failure patients	ICM and NICM with LVEF of 30% or less	CRT-D vs ICD alone	Early intervention with CRT-D in patients with a left bundle-branch block was associated with a significant reduction in heart failure events
Bristow et al. (2004) [11]	To evaluate the use of prophylactic cardiac resynchronization therapy with or without a defibrillator to reduce the risk of death and hospitalization among patients with advanced chronic heart failure	ICM and NICM with QRS intervals of at least 120 msec	CRT vs CRT-D vs optimal pharmacologic therapy	As compared with optimal pharmacologic therapy alone, CRT with pacemaker decreased the risk of death as did CRT with a pacemaker-defibrillator
Ruschitzka et al. (2013) [12]	To evaluate the effect of CRT in patients with heart failure	Heart failure with an LVEF of 35% or less, a QRS duration of less than 130 msec, and echocardiographic evidence of left ventricular dyssynchrony	CRT-D vs optimal medical therapy alone	In patients with systolic heart failure and a QRS duration of less than 130 msec, CRT does not reduce the rate of death or hospitalization for heart failure and may increase mortality
Al-Khatib et al. (2018) [13]	To review 2018 guidelines for the treatment of heart failure in the prevention of SCD	All NYHA Classification	Several therapy options discussed, including the use of ICD	Significant variation was observed in the clinical effectiveness of ICDs between patients with an LVEF ≤35% and an LVEF >35%
Ponikowski et al. (2016) [14]	To review 2016 guidelines for the diagnosis and treatment of acute and chronic heart failure	All NYHA Classification	Several therapy options discussed, including the use of ICD	Significant variation was observed in the clinical effectiveness of ICDs between patients with an LVEF ≤35% and an LVEF >35%
Priori et al. (2015) [15]	To review 2015 European guidelines for the management of patients with ventricular arrhythmias and the prevention of SCD	All NYHA Classification	Several therapy options discussed, including the use of ICD	Significant variation was observed in the clinical effectiveness of ICDs between patients with an LVEF ≤35% and an LVEF >35%
Yancy et al. (2017) [16]	To review 2017 update for the guidelines in the management of heart failure	All NYHA Classification	Several therapy options discussed, including the use of ICD	Significant variation was observed in the clinical effectiveness of ICDs between patients with an LVEF ≤35% and an LVEF >35%
Moss et al. (2002) [17]	To evaluate the effect of an implantable defibrillator on survival in patients with HF	HF with prior history of MI and LVEF of 30% or less	ICD vs medical therapy alone	Although prophylactic implantation of a defibrillator improves survival, inappropriate shocks may increase the risk of death due to heart failure progression
Poole et al. (2008) [18]	To determine the prognostic importance of defibrillator shocks in patients with heart failure	All NYHA Classification	ICD vs no ICD	Those who receive inappropriate shocks for any arrhythmia have a substantially higher risk of death than similar patients who do not receive such shocks

Several studies have investigated the role of ICDs in patients with NICM who are receiving effective quadruple therapy for heart failure. One such clinical trial is a retrospective study that analyzed data from the Medicare database [4]. The article found that ICD implantation was associated with a significant reduction in all-cause mortality in patients with NICM who were receiving optimal medical therapy [4]. However, the study was limited by its retrospective design and the lack of information on the cause of death. Another article provides comparable evidence, in which the study concluded that ICDs, in addition to the use of angiotensin-converting enzyme (ACE inhibitors) and beta-blockers, in patients with severe, non-ischemic dilated cardiomyopathy significantly reduced the risk of sudden death from arrhythmia [5]. Additionally, the DEFINITE trial evaluated the use of ICD therapy in NICM patients with a low ejection fraction (EF) who were already receiving optimal medical therapy (including beta-blockers, ACE inhibitors or angiotensin receptor blockers (ARBs), and diuretics). The study found that ICD therapy significantly reduced the risk of sudden cardiac death (SCD) in this patient population but did not have a significant impact on overall mortality [6].

In contrast, a randomized controlled trial, the DANISH trial, found that ICD implantation did not significantly reduce all-cause mortality in patients with NICM who were receiving optimal medical therapy. The study included 1,520 patients with NICM and left ventricle ejection fraction (LVEF) ≤35% who were receiving optimal medical therapy. The authors concluded that ICD implantation did not significantly reduce all-cause mortality or rate of hospitalization for cardiovascular reasons [7]. Similarly, a recent meta-analysis reviewed data from eight randomized controlled trials and found that ICD implantation significantly reduced the risk of SCD but did not significantly reduce all-cause mortality in patients with NICM who were receiving optimal medical therapy. The meta-analysis also found that the benefit of ICD implantation was greater in patients with lower LVEF and in those with non-sustained ventricular tachycardia [8,9].

The MADIT-CRT trial was a randomized controlled trial that evaluated the use of cardiac resynchronization therapy (CRT) with or without an ICD in patients with ischemic cardiomyopathy (NYHA functional class I or II) or NICM (NHYA functional class II only), an LVEF of 30% or less, and a prolonged QRS duration (≥130 msec). Data provided evidence that treatment with CRT-D is associated with a significant long-term survival benefit in patients with mild heart failure who have an LVEF of 30% or less and a left bundle-branch block (LBBB). However, no clinical benefit was observed in patients with mild heart failure without LBBB [10]. Another article with similar findings is that by Bristow et al. (2004) [11]. The study describes a randomized clinical trial evaluating the efficacy of CRT with or without an ICD in patients with advanced chronic heart failure due to ischemic or non-ischemic cardiomyopathies. The trial enrolled patients with NYHA class III or IV heart failure, LVEF ≤35%, and a QRS duration of ≥120 msec. Participants were randomized to receive either CRT alone or CRT with an ICD. The primary endpoint was all-cause mortality, but the trial also evaluated secondary endpoints, including the incidence of hospitalization for heart failure, quality of life, and left ventricular function. Results demonstrated a significant reduction in all-cause mortality in the CRT with ICD group compared to the CRT alone group. The study also showed a reduction in hospitalization for heart failure and an improvement in quality of life in the CRT with ICD group compared to the CRT alone group [11]. Overall, the trial provided strong evidence for the efficacy of CRT with an ICD in improving outcomes in patients with advanced chronic heart failure, supporting the use of this therapy in clinical practice.

In comparison, another study evaluating the use of CRT-D in patients with heart failure found no benefit for those receiving stable medical therapy, a reduced LVEF, and a QRS duration of <120 msec. In this patient population, this therapeutic approach did not reduce the rate of death from all causes or hospitalization with a possible increase in their mortality [12]. Several studies have analyzed the guidelines for the management of patients with ventricular arrhythmias and the prevention of SCD. In terms of treatment, these guidelines recommend the use of ICD therapy for primary prevention in patients with certain high-risk conditions, such as NICM with reduced LVEF, recent myocardial infarction (MI) with reduced LVEF, and undergoing coronary revascularization procedure with reduced LVEF [13-16]. The studies in these articles suggest that ICDs in this population might prolong life expectancy, particularly after optimal medical therapy has failed to improve LVEF (>35%).

Aside from considering the potential benefits of ICDs placement in patients with heart failure, potentially drawbacks may also be encountered. A few studies found that those patients who received an ICD for primary prevention may have later received therapeutic shocks for any arrhythmia, resulting in a substantially higher risk of death than similar patients who did not receive such shocks. Although a decreased rate of SCD still occurred after ICD placement, it was concluded that the most common cause of death among these patients was progressive heart failure [15,17,18].

## Conclusions

The summary of this literature review is to assess and explore the effectiveness of ICD placement in patients with NICM in view of evidence-based and goal-directed effective quadruple therapy. RCM and HCM treatment guidelines do not involve quadruple therapy as their ejection fraction is >35% which is one of the criteria for ICD placement and was not explored in this study, thereby focusing on the DCM type of NICM with its effective management with quadruple therapy and the usage of ICD for the reduction in mortality and risk of SCD. Based on multiple kinds of literature reviewed, it was concluded that ICD decreases the risk of SCD but does not decrease mortality as multiple trials and meta-analyses predicted. ICD use in NICM patients with an EF ≤35% getting successful quadruple treatment for HF is still debatable and needs additional research in this regard. Overall, individual considerations including the patient's clinical history, social economic status, and risk factors should go into the decision to adopt ICD therapy in NICM patients.
